# Cortical mechanisms of sensory trick in cervical dystonia

**DOI:** 10.1016/j.nicl.2023.103348

**Published:** 2023-02-11

**Authors:** Nicoletta Manzo, Giorgio Leodori, Giulia Ruocco, Daniele Belvisi, Shabbir Hussain I. Merchant, Giovanni Fabbrini, Alfredo Berardelli, Antonella Conte

**Affiliations:** a Department of Human Neurosciences, Sapienza University of Rome, Viale dell'Università 30, Rome 00185, Italy; b IRCCS San Camillo Hospital, Via Alberoni 70, Venice 30126, Italy; c IRCCS Neuromed, Via Atinense 18, Pozzilli, IS 86077, Italy; d Beth Israel Deaconess Medical Center/ Harvard Medical School, Boston, USA

**Keywords:** Sensory trick, Dystonia, Event-related desynchronization, Sensorimotor cortex, Posterior parietal cortex

## Abstract

Patients with cervical dystonia (CD) often show an improvement in dystonic posture after sensory trick (ST), though the mechanisms underlying ST remain unclear. In this study, we aimed to investigate the effects of ST on cortical activity in patients with CD and to explore the contribution of motor and sensory components to ST mechanisms. To this purpose, we studied 15 CD patients with clinically effective ST, 17 without ST, and 14 healthy controls (HCs) who mimicked the ST. We used electroencephalographic (EEG) recordings and electromyography (EMG) data from bilateral sternocleidomastoid (SCM) muscles. We compared ST-related EEG spectral changes from sensorimotor and posterior parietal areas and EMG power changes between groups. To better understand the contribution of motor and sensory components to ST, we tested EEG and EMG correlates of three different conditions mimicking ST, the first without skin touch (“no touch” condition), the second without voluntary movements (“passive” condition), and finally without arm movements (“examiner touch” condition).

Results showed ST-related alpha desynchronization in the sensorimotor cortex and theta desynchronization in the sensorimotor and posterior parietal cortex. Both spectral changes were more significant during maneuver execution in CD patients with ST than in CD patients without ST and HCs who mimicked the ST. Differently, the “no touch”, “passive”, or “examiner touch” conditions did not show significant differences in EEG or EMG changes determined by ST execution/mimicking between CD patients with or without ST. A higher desynchronization within alpha and theta bands in the sensorimotor and posterior parietal areas correlated with a more significant activity decrease in the contralateral SCM muscle, Findings from this study suggest that ST-related changes in the activity of sensorimotor and posterior parietal areas may restore dystonic posture and that both motor and sensory components contribute to the ST effect.

## 1 Introduction

Sensory trick (ST) is a characteristic feature of idiopathic cervical dystonia (CD) characterized by an improvement in dystonic symptoms when the patient touches the affected body segment (Patel et al., 2014). In CD, ST reduced or abolished excessive electromyographic (EMG) activity in neck muscles contributing to the dystonic posture (Wissel et al., 1999, Schramm et al., 2004). Recent studies have suggested that CD may be due to a dysfunction in a network of brain regions, including the basal ganglia, brainstem, cerebellum, and cortical areas. Various pathophysiological mechanisms have been proposed for dystonia in CD, including abnormal sensorimotor integration (Abbruzzese and Berardelli, 2003, Jinnah and Hess, 2018, Shakkottai et al., 2017, Conte et al., 2019a).

ST may produce its clinical effects by interfering with CD pathophysiology. A study investigating intracortical excitability with transcranial magnetic stimulation (TMS) protocols in CD patients showed that ST modulates excessive facilitation in the primary motor cortex (M1) (Amadio et al., 2014). One study performed on electroencephalographic (EEG) recording in two CD patients with deep brain stimulation implanted in the globus pallidus internus (GPi) reported that ST was associated with oscillatory activity desynchronization in the theta-mu band in the GPi and sensorimotor cortex (Tang et al., 2007). Neuroimaging studies showed that ST in patients with CD was associated with sensorimotor and parietal cortex activation (Naumann et al., 2000, Sarasso et al., 2020). Overall, the above observations suggest that ST may interfere with abnormal cortical activity related to dystonic posture, possibly by transiently modulating sensorimotor integration. However, the cortical correlates of ST effects and how ST-related motor output (voluntary arm movement) and sensory input (skin touch) interact to determine ST effects are still unknown.

The present study aimed to investigate cortical mechanisms underlying ST in patients with CD. We investigated ST effects on sensorimotor and parietal oscillatory activity (Naumann et al., 2000, Sarasso et al., 2020) as measured by EEG spectral changes, as well as the effects of ST on EMG activity in neck muscles affected by dystonia. If ST’s beneficial effects on CD are due to cortical mechanisms, then we would expect maneuver to be associated with different EEG changes in the theta-mu band in patients with ST compared to patients without ST (Chen et al., 2006, Neumann et al., 2017). To investigate the contribution of motor and sensory components to ST, we tested the EEG and EMG correlates of three different conditions mimicking ST, the first without skin touch (“no touch” condition), the second without voluntary movements (“passive” condition), and finally without arm movements (“examiner touch” condition). Finally, to investigate whether EEG and EMG changes specifically reflect ST mechanisms rather than movement per se or touch-related effects, data of patients with ST were compared to those obtained from a sample of matched healthy controls (HCs).

## 2 Methods

### 2.1 Participants

The study included 15 CD patients with effective ST. Data from these patients were compared to those obtained from 17 CD patients without ST and 14 HCs. Patients without ST and HCs were age and gender-matched to those with ST. Patients were enrolled in the outpatient clinic of the Sapienza University of Rome. All participants provided written informed consent, and the experiments were carried out following The Code of Ethics of the World Medical Association (Declaration of Helsinki). The institutional, local ethics committee approved the study protocol. Clinical evaluation was performed by a movement disorders specialist using the Toronto Western Spasmodic Torticollis Rating Scale (TWSTRS) (Consky and Lang 1994). All patients had laterocollis (17 right, 15 left) and 16 had dystonic tremors. ST was also investigated, and patients who reported “complete, partial, or limited effect of sensory trick” as assessed by the specific TWSTRS item (Consky and Lang 1994) were included in the ST group only when clinical observation confirmed the presence of ST. Exclusion criteria were secondary forms of dystonia, other neurological diseases, botulinum toxin injection in the three months preceding the testing session, and the intake of drugs interfering with central nervous system activity.

### 2.2 EMG and EEG recording

Surface EMG activity was recorded from the bilateral sternocleidomastoid (SCM) muscles and bicep brachii muscle of the side used in the task using 9-mm-diameter Ag-AgCl surface electrodes. Both active and reference electrodes were placed 2 cm apart in the muscle belly, with the ground electrode over the forehead. EMG signals were recorded (D360 amplifier, Digitimer Ltd, Welwyn Garden City, UK), amplified (x1000), filtered (band-pass 3–3000 Hz), and sampled at 5 kHz using a 1401 power analog-to-digital converter and Signal 6 software (Cambridge Electronic Design, Cambridge, UK).

EEG was recorded with a 32-channel system (Bittium, NeurOne, Finland) connected to a 32-channel EEG cap with 32 Ag/AgCl electrodes (EASYCAP, Herrsching, Germany). Recording electrodes were arranged according to the 10–20 international EEG system and included Fp1-Fp2-AFz-F7-F3-Fz-F4-F8-FC5-FC1-FCz-FC2-FC6-T7-C3-Cz-C4-T8-TP9-CP5-CP1-CP2-CP6-TP10-P7-*P*3-Pz-*P*4-P8-O1-O2-Iz. Electrodes were POz‐referenced and FPz‐grounded. Impedance was kept below 5 kΩ. Surface electrodes were placed over the skin area involved in ST to record the ‘touch’ event.

### 2.3 Experimental paradigm

Experiment 1 – ST effects: patients with ST were asked to perform the arm movement that produced the ST, whereas patients without ST and HCs were asked to mimic the ST by touching their faces. For patients without ST and HCs, the arm used for mimicking the ST was chosen to have a similar right-to-left proportion to patients with ST. HCs were also asked to mimic CD by rotating the head approximately 30–45 degrees, and the side of the head rotation in HCs was chosen to have a similar right-to-left proportion to patients with ST. HCs were also asked to move their heads back to the neutral position after the touch to mimic the ST effect.

Experiment 2 – No-touch condition: patients were asked to perform the arm movement that produced/mimicked the ST, but an object held by a mechanical arm about 2 cm away from the head/face prevented the subject from touching the head/face.

Experiment 3 – Passive condition: the patient’s arm was passively moved by the examiner to simulate ST/mimicked ST.

Experiment 4 – Examiner touch condition: with the patient at rest, the examiner stood behind the patient and touched the patient’s face in the same place used for the ST/mimicked ST.

**Fig. 1:**
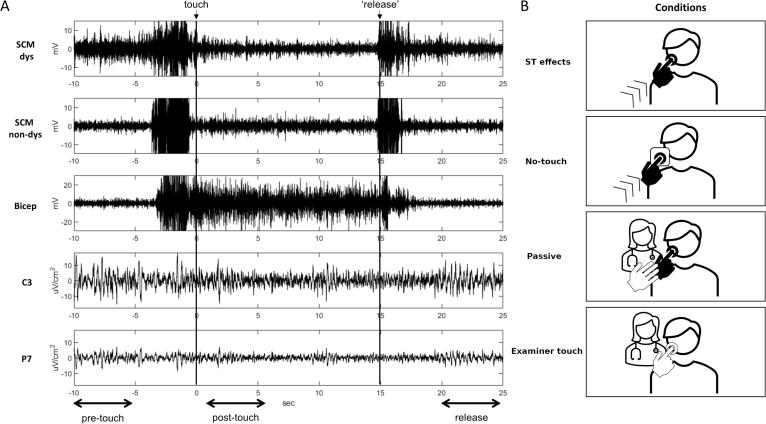
A: trial from one representative patient with a sensory trick (ST) in experiment 1. EMG from right dystonic (SCM dys) and left non-dystonic sternocleidomastoid (SCM non-dys), from bicep brachii (Bicep), and EEG from left sensorimotor (C3) and posterior parietal cortex (P7). Arrows: time windows of interest. B: experimental conditions.

Both patients and HCs underwent experiment 1, whereas only patients participated in experiments 2, 3, and 4. Each experiment consisted of 30 trials separated by a 15-s intertrial interval. The examiner gave verbal cues to indicate the beginning of each trial, but after the cue participants spontaneously decided when to initiate the task. Another examiner was involved in “passive” and “examiner touch” conditions. Participants and the examiner performed a self-paced ST/mimicked ST, maintained the touch for 15 s post-touch, and then released the touch to return to the rest position. Continuous EMG-EEG co-registration was performed during the experimental procedures. An electrode was placed over the head/face spot being touched to record the touch event on the EMG and EEG recordings. The order of experimental conditions was pseudo-randomized across participants (Fig. 1).

### 2.4 Signal analysis

EMG and EEG analyses were performed using MATLAB toolboxes (EEGLAB, Fieldtrip) (Oostenveld et al., 2011). EMG signal was down-sampled to 500 Hz, band-pass filtered between 20 and 220 Hz, and notch-filtered (zero-phase, 4th-order Butterworth). High pass filtering above 20 Hz limits contamination by movement artifact and volume conduction without losing information on dystonic activity below 20 Hz frequency (Grosse et al., 2004, Foncke et al., 2007).

EEG signal was down-sampled to 500 Hz, band-pass filtered between 1 and 100 Hz, and notch-filtered (zero-phase, 4th-order Butterworth). Continuous EMG and EEG recordings were epoched and time-locked from −15 s before to 55 s after the touch events. Epochs containing large EMG or EEG artifacts were rejected by visual inspection. FastICA was used to clean EEG epochs from muscle and eye movements and residual line noise artifacts. We computed the current source density of cleaned EEG epochs to obtain non-referential high spatial resolution distribution of surface voltage of the scalp (Kayser and Tenke 2015).

Further analyses were carried out on the EMG signal from the SCM muscle responsible for head-turning (i.e., the dystonic SCM in CD patients and the one activated to mimic CD in HCs) and from contralateral EEG channels. Also, EMG and EEG analysis was carried out on the following three intervals of interest: from −10 to −5 s before the touch (pre-touch), from 0.5 to 5.5 s after the touch (post-touch interval), and from 5 to 10 s after release (release) (Fig. 1A).

Fast Fourier transform (FFT) with Hanning tapering was used to obtain EMG and EEG power spectral density values from 1 to 100 Hz over the pre-touch interval. Complex Morlet wavelet convolution (Cohen 2019) was used to compute time–frequency power spectra from 1 to 100 Hz in the EMG signal, and from 1 to 50 Hz in the EEG, as described in a previous study (Leodori et al., 2021). Time-frequency power spectra were decibel normalized to the average value of the pre-touch interval to obtain touch-related power modulation values. We computed mean EMG power and modulation values by averaging EMG spectra from 1 to 100 Hz. Similarly, we computed mean EEG power values and event-related spectral perturbation (ERSP) relative to the touch by averaging across the theta (4–7 Hz), alpha (8–12 Hz), beta (13–30 Hz), and gamma (31–45 Hz) frequency bands, and across electrodes over the sensorimotor cortex (C3/4, CP1/2) and posterior parietal cortex (PPC) (P3/4, P7/8).

### 2.5 Statistical analysis

The Kruskal-Wallis test was conducted to investigate differences in age between patients with ST, patients without ST, and HCs groups. Chi-square tests of homogeneity were used to investigate differences between the three groups in the distribution of gender, side of the arm used in the task, and to check the proportion of patients with dystonic tremor between patients with and without ST. An unpaired *t*-test was run to investigate differences in TWSTRS scores between patients with and without ST.

Data normality was tested for EMG and EEG variables by skewness and kurtosis values. The assumption of normality was satisfied for baseline EMG and for EMG power modulation values after the removal of one outlier (2.5 standard deviations (SDs); results were not affected by removing the outlier), but not for baseline EEG and ERSP values.

Experiment 1: For baseline EMG power, one-way analysis of variance (ANOVA) was used to investigate the effect of the factor group (patients with ST, patients without ST, and HCs) on EMG power pre-touch. To evaluate the effect of ST, mixed-design ANOVAs were used to test the effect of the factors time (post-touch, release) and group (patients with ST, patients without ST, and HC) on EMG power modulation.

For baseline EEG power, false discovery rate (FDR)-corrected Kruskal-Wallis tests with Dunn's post hoc comparisons were conducted to investigate differences in EEG power pre-touch between groups (patients with ST, patients without ST, HCs) across the different frequency bands and area on EEG power pre-touch. To evaluate the effect of ST, we performed a MANOVA with ERSP at different frequency bands as dependent variables (theta, alpha, beta, gamma), and group (patients with ST, patients without ST, and HCs), time (post-touch, release) and cortical area (sensorimotor vs posterior parietal cortex) as independent variables. Since MANOVA is robust to deviations from normality (Weinfurt 1995), we performed a parametric test regardless of the non-normal distribution of ERSP values. FDR correction was applied to MANOVA post hoc multiple univariate interaction effects.

Spearman's correlation assessed the relationship between ERSP associated with ST and EMG power modulation considering an FDR correction for multiple correlations. Outliers in correlation analysis were defined as data points with standardized residuals greater than ± 3 standard deviations.

Experiments 2, 3, and 4: Mixed-design ANOVAs used for experiment 1 analysis were repeated to compare EMG power modulation and ERSP between patients with ST and patients without ST in the no touch, passive, and examiner touch conditions.

## 3 Results

All the study participants completed the experimental procedure. Data are expressed as mean *±* SD unless otherwise indicated. No difference was found between patients with and without ST and HCs groups either in age median values (62.0 vs 60.0 vs 59.5, χ2(2) = 0.12, p = 0.57), or in the distribution of gender (9 F (60 %) vs 10 F (59 %) vs 7 F (50 %); χ2(2) = 0.35, p = 0.84), side of the arm used in the task (9 R (75 %) vs 12 R (71 %) vs 10 R (71 %); χ2(2) = 0.73, p = 0.96). There were no significant differences in the proportions of patients with dystonic tremor between patients with and without ST (7 (47 %) vs 9 (53 %); χ2(1) = 0.13, p = 0.72). TWSTRS score was normally distributed, as assessed by Shapiro-Wilk's test (p > 0.05) and was not significantly different between patients with and without ST (20.20 ± 9.31 vs 23.59 ± 12.57, t(30) = 0.86, p = 0.40).

### 3.1 Experiment 1 – ST effect on muscular and cortical activity

**Fig. 2:**
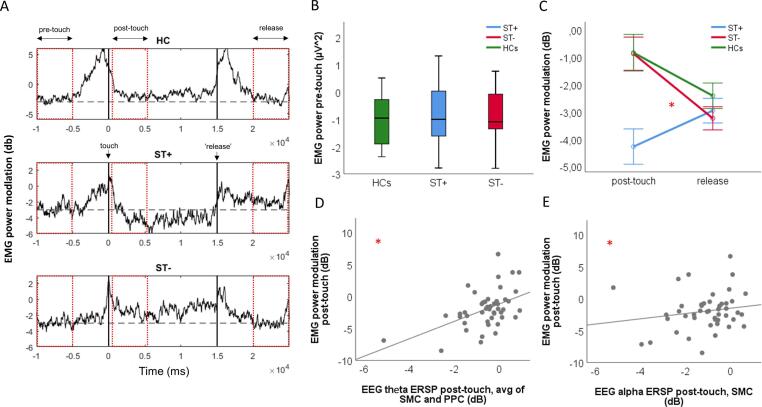
A: Group average EMG power modulation (mean 1–100 Hz) from the sternocleidomastoid muscle (SCM) of healthy controls (HC), and of cervical dystonia (CD) patients with (ST + ) and without sensory trick (ST-). Dashed lines: mean pre-touch values. Rectangles highlight time windows of interest. B: interquartile (boxes), minimum and maximum (whiskers), median (horizontal lines). C: bars = SEM *: p < 0.05. D, E: Event-related spectral perturbation (ERSP); dots = individual CD patients.

Mixed-design ANOVAs on EMG power modulation in experiment 1 showed significant time*group interaction. It be explained by the significantly lower EMG power at post-touch than at release in ST patients, compared to the significantly higher values at post-touch than at release in patients without ST and in HCs. Also, we found significantly higher EMG power reduction post-touch in patients with ST as compared to patients without (p < 0.001) and HCs (p = 0.001), but no significant differences at release between patients with ST and patients without ST (p = 0.651) and HCs (p = 0.408) (Fig. 2).

**Fig. 3:**
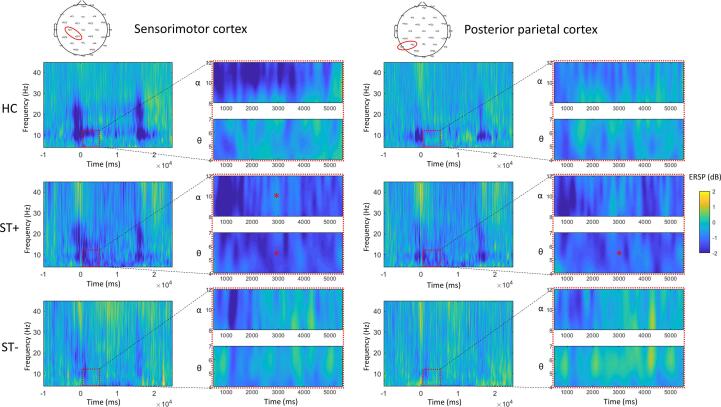
Event-related spectral perturbation (ERSP) averaged across electrodes over the sensorimotor (C3/4, CP1/2) (left) and posterior parietal cortex (*P*3/4, P7/8) (right) contralateral to dystonic/activated sternocleidomastoid muscle in cervical dystonia (CD) patients with a sensory trick (ST + ), without (ST-), and in healthy controls (HCs); brackets: frequency bands of interest (zoomed in for the alpha and theta band); arrows: time windows of interest. Rectangles highlight differences at post-touch. *: p < 0.05.

**Fig. 4:**
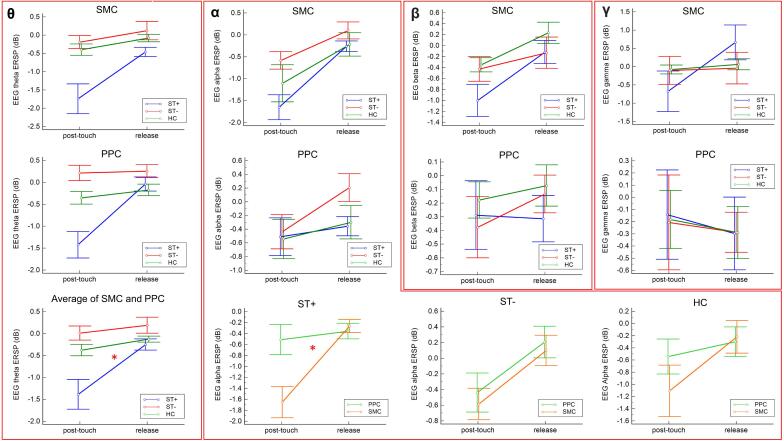
Event-related desynchronization in the theta, alpha, beta, and gamma band in sensorimotor cortex (SMC) or posterior parietal cortex (PPC) in CD patients with ST (ST + ), without ST (ST-) and in healthy controls (HCs). *:p < 0.05; bars = SEM.

Baseline theta power median values were significantly different between groups at the sensorimotor cortex level. In contrast, no other significant differences in baseline EEG power were found in the other frequency bands and areas. Post hoc analysis revealed significantly lower baseline theta power at sensorimotor cortex level in HCs (-1.52) compared to both CD patients with ST (-1.12) and without (-1.04), but no significant differences between CD groups. MANOVA on ERSP showed a significant group*time*area interaction on the combined dependent variables theta, alpha, beta, and gamma ERSP. Univariate analyses showed a significant group*time*area interaction only on alpha ERSP, a significant group*time interaction only on theta but not for alpha, beta, or gamma ERSP, and a significant effect of factor group only on theta and alpha ERSP. Post hoc analysis on alpha ERSP showed a significant area*time interaction only in patients with ST explained by significantly greater event-related alpha desynchronization at post-touch compared to release only at sensorimotor cortex level, but not a PPC level. Post hoc analyses on theta ERSP values averaged between sensorimotor cortex and PPC showed a significant effect of factor time only in patients with ST, explained by a significantly greater theta desynchronization at post-touch compared to release. In contrast, no significant effect of time was found for patients without ST and HCs (see Table 1 for detailed results) (Fig. 3, Fig. 4). Finally, post hoc analyses on the main effect of group (independent of time and areas), demonstrated more theta desynchronization in patients with ST compared to patients without (p < 0.001) and HCs (p = 0.008), but no significant differences between patients without ST and HCs (p = 0.084). Also, we found more alpha desynchronization in patients with ST compared to patients without (p = 0.006). However, no significant differences compared to HCs (p = 0.420), and only a trend toward significant differences between patients without ST and HCs (p = 0.055).

**Table 1:** Main results.

**Factor**	**DV**	**Level**	**df**	**F(λ)/H**	**p/p-adj.**
**EXPERIMENT 1**					
**1-WAY ANOVA**					
Group	EMG pwr. Pre-touch		2;43	0,139	0,871
**Mixed ANOVA**					
Time * Group	EMG pwr. Modulation		2;43	7,79	**0,001**
Time	EMG pwr. Modulation	ST+	1;14	10.55	**0,008**
Time	EMG pwr. Modulation	ST-	1;16	4,71	**0,023**
Time	EMG pwr. Modulation	HCs	1;13	10,72	**0,006**
**KRUSKALL-WALLIS – FDR-corrected**					
Group	EEG θ pwr pre-touch	SMC	2	15,25	**0,008**
Group	EEG α pwr pre-touch	SMC	2	3,94	0,159
Group	EEG β pwr pre-touch	SMC	2	5,93	0,083
Group	EEG γ pwr pre-touch	SMC	2	8,64	0,052
Group	EEG θ pwr pre-touch	PPC	2	6,46	0,08
Group	EEG α pwr pre-touch	PPC	2	2,66	0,264
Group	EEG β pwr pre-touch	PPC	2	4,57	0,136
Group	EEG γ pwr pre-touch	PPC	2	6,58	0,08
post hoc – Dunn’s pairwise comparisons					
Group (ST + vs ST-)	EEG θ pwr pre-touch	SMC			0,086
Group (ST + vs HCs)	EEG θ pwr pre-touch	SMC			**0,002**
Group (ST- vs HCs)	EEG θ pwr pre-touch	SMC			**0,002**
**MANOVA**					
Area * Time * Group	ERSP θ,A,B,G		8;80	2,916(0,599)	**0,007**
3-way univariate ANOVAs					
Area	ERSP θ		1;43	6,767	**0,013**
	ERSP α		1;43	8,310	**0,006**
	ERSP β		1;43	0,577	0,452
	ERSP γ		1;43	1,565	0,218
Time	ERSP θ		1;43	12,404	**0,001**
	ERSP α		1;43	10,902	**0,002**
	ERSP β		1;43	7,929	**0,007**
	ERSP γ		1;43	1,135	0,293
Group	ERSP θ		2;43	11,164	**<0,001**
	ERSP α		2;43	4,377	**0,019**
	ERSP β		2;43	1,253	0,296
	ERSP γ		2;43	0,010	0,990
Area * Time	ERSP θ		1;43	2,446	0,125
	ERSP α		1;43	13,246	**0,001**
	ERSP β		1;43	8,472	**0,006**
	ERSP γ		1;43	6,121	0,017
Area * Group	ERSP θ		2;43	2,565	0,089
	ERSP α		2;43	1,336	0,274
	ERSP β		2;43	0,984	0,382
	ERSP γ		2;43	0,010	0,990
Time * Group	ERSP θ		2;43	4,338	**0,019**
	ERSP α		2;43	0,083	0,920
	ERSP β		2;43	0,142	0,868
	ERSP γ		2;43	1,085	0,347
Area * Time * Group	ERSP θ		2;43	0,097	0,908
	ERSP α		2;43	4,068	**0,024**
	ERSP β		2;43	2,332	0,109
	ERSP γ		2;43	2,913	0,065
post hoc for 3-way interaction on ERSP α					
Area * Time	ERSP α	ST+	1;14	9,679	**0,008**
Area * Time	ERSP α	ST-	1;16	0,052	0,823
Area * Time	ERSP α	HCs	1;13	3,762	0,074
Time	ERSP α	ST+,SMC	1;14	17,439	**0,001**
Time	ERSP α	ST+,PMC	1;14	0,259	0,619
post hoc for Time * Group interaction on ERSP θ					
Time	ERSP θ, mean (SMC,PPC)	ST+	1;14	14,600	**0,002**
Time	ERSP θ, mean (SMC,PPC)	ST-	1;16	0,412	0,530
Time	ERSP θ, mean (SMC,PPC)	HCs	1;13	3,130	0,100

**CONTROL EXPERIMENTS**					
**Mixed ANOVA**					
Exp.2: Group * Time	EMG pwr. Modulation		1;30	0,190	0,89
Exp.3: Group * Time	EMG pwr. Modulation		1;30	0,27	0,608
Exp.4: Group * Time	EMG pwr. Modulation		1;30	1,39	0,249
**MANOVA**					
Exp.2: Group * Time * Area	ERSP θ,α,β,γ		4;27	2,2(0,73)	0,1
Exp.3: Group * Time * Area	ERSP θ,α,β,γ		4;27	0,22(0,97)	0,925
Exp.4: Group * Time * Area	ERSP θ,α,β,γ		4;27	1,21(0,83)	0,332

Abbreviations: λ: Wilks’ Lambda; H: Kruskall-Wallis’s H. ST+: patients with a sensory trick. ST-: patients without Sensory Trick. HCs: healthy controls. ERSP: Event-related Spectral Perturbation. Θ: theta band, α: alpha band, β: beta band, γ: gamma band. SMC: sensorimotor cortex. PPC: Posterior Parietal Cortex.

In CD patients at post-touch there was a significant positive correlation between sensorimotor alpha ERSP and parietal-sensorimotor theta ERSP values (rs(32) = 0.665, p < 0.001), and both ERSP measures showed a significant positive correlation with EMG power modulation (alpha: rs(32) = 0.359, p = 0.044; theta: rs(32) = 0.576, p = 0.001) (Fig. 2). There was one outlier in the correlation analysis between parietal-sensorimotor theta ERSP and EMG power modulation. The analysis repeated without the outlier yielded similar results (rs(31) = 0.538, p = 0.002).

### 3.2 Experiments 2, 3, and 4 – Motor and sensory components contribution to ST

Mixed-design ANOVAs comparing EMG power modulation at post-touch and release between patients with and without ST showed non-significant group*time interactions in the passive condition, no touch condition, and examiner touch condition.

Similarly, MANOVAs on the combined dependent variables theta, alpha, beta, and gamma ERSP showed non-significant group*time*area interactions in the passive condition, no touch condition, and examiner touch condition (Table 1).

### 3.3 Control analyses

Unpaired t-tests were run to compare EMG power modulation, and the Mann-Whitney *U* test was performed to compare ERSP between patients with and without a dystonic head tremor to control for possible confounding effects. There were no significant differences between CD patients with tremor and without in post-touch EMG power modulation (-2.39 ± 2.88 db vs −2.56 ± 3.50 db, t(30) = 0.15, p = 0.881), post-touch alpha ERSP on the sensorimotor cortex (median −0.66 vs −1.27 db, U = 170, p = 0.119), or post-touch theta ERSP on the sensorimotor and PPC (median −0.34 vs −0.34 db, U = 150, p = 0.423).

To investigate the side specificity of ST effects on EEG data, we performed a MANOVA on ERSP values using EEG channels ipsilateral to the dystonic SCM. Non-significant group*time*area interaction effect on the combined dependent variables theta, alpha, beta, and gamma ERSP (F(4, 27) = 1.922, p = 0.136, Wilks’ Λ = 0.778).

**Fig. 5:**
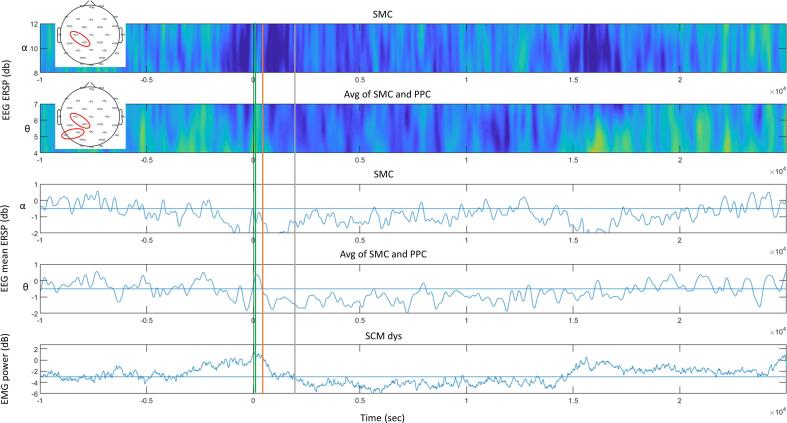
Top: group average alpha band event-related spectral perturbation (ERSP) in patients with a sensory trick (ST + ) from the sensorimotor cortex (SMC) contralateral to the dystonic sternocleidomastoid muscle (SCM dys); theta band ERSP from the average of SCM and posterior parietal cortex (PPC); blue: desynchronization, yellow: synchronization. Middle: EEG mean alpha and theta ERPS. Bottom: ST + group average EMG power modulation from the SCM dys. Lines: touch event (black), onset of alpha (green) and theta (orange) band desynchronization, onset of EMG power reduction (grey). (For interpretation of the references to colour in this figure legend, the reader is referred to the web version of this article.)

To investigate whether changes in post-touch EEG activity may reflect sensory inflow related to head posture normalization rather than mechanisms of ST, we compared the time course of average post-touch modulation between EMG and ERSP. Visual comparison between ST-related EMG and EEG modulations showed that while mean EMG power reduction began around 1 s post-touch, mean theta and alpha band desynchronization onset occurred simultaneously with the touch or even slightly before (Fig. 5).

## 4 Discussion

The present study provided novel information on the neurophysiological mechanisms of ST in CD. Alpha desynchronization in the sensorimotor cortex and theta desynchronization in the sensorimotor and posterior parietal cortex were more evident in CD patients during ST, than in CD patients without ST and in HCs when they mimicked the ST. We also found that sensorimotor alpha and sensorimotor-parietal theta spectral changes correlated. A higher desynchronization within these bands and areas correlated with a larger contralateral SCM activity decrease post-touch. In the experiments simulating the ST (no touch, passive, and examiner touch conditions) we found no differences in post-touch EEG and EMG changes between CD patients with and without ST. The results suggest that alpha activity in the sensorimotor cortex and theta activity in a parietal-sensorimotor network contralateral to the dystonic SCM muscle play an essential role in the ST mechanisms.

We adopted several methodological precautions to avoid possible confounding. There was a similar age and gender distribution between groups, which excluded demographic-related bias. Movement onset and movement execution were self-paced to reproduce real-world ST-related features and effects. The intertrial interval was randomized to limit possible habituation effects, and the order of experimental conditions was pseudo-randomized among participants to limit experimental duration bias. We limited possible confounding due to hemispheric dominance since 30 out of 32 patients and all HCs were right-handed. Moreover, the arm used in the task in HCs and patients without ST was matched with patients with ST. We asked HCs to rotate their neck to simulate the dystonic posture, limiting possible confounding due to different baseline head positions and SCM activation. Finally, we can exclude tremor-related bias since we found no differences in ST effects on EMG and EEG activity between patients with and without dystonic tremor.

The observation that sensorimotor alpha desynchronization and parietal-sensorimotor theta desynchronization were more evident on the side contralateral to dystonic SCM in patients with ST, compared to patients without ST and HCs mimicking the ST, suggests that these spectral changes reflect cortical activity specifically associated with ST. Moreover, since post-touch alpha and theta desynchronization started before the EMG power reduction, we can exclude the possibility that sensorimotor and PPC activity changes are secondary to reduced SCM activity.

Desynchronization in the alpha and theta band reflects the attenuation of the activity of a neuron population firing simultaneously in this frequency range (Neuper et al., 2006, Pfurtscheller and Klimesch, 1991, Pfurtscheller and Klimesch, 1992, Pfurtscheller and Lopes da Silva, 1999). The finding that ST is associated with a desynchronization in the alpha band specifically taking place in the sensorimotor area contralateral to the dystonic SCM muscle is in line with previous observations showing that alleviation of dystonia during ST was accompanied by desynchronization in the alpha band (Tang et al., 2007). This observation suggests a link between ST effects and changes in sensorimotor cortex activity (Tang et al., 2007, Naumann et al., 2000, Sarasso et al., 2020).

We also found a specific association between ST and theta desynchronization over the parietal-sensorimotor cortical area. One hypothesis to explain ST-related theta desynchronization is that it may result from the modulation of abnormal theta oscillations at the subcortical level (Hutchison et al., 2004, Halje et al., 2019). Following this hypothesis, several recent studies in CD patients investigating local field potentials have highlighted increased theta activity synchronization in the GPi and subthalamic nucleus (Chen et al., 2006, Neumann et al., 2012, Neumann et al., 2017, Geng et al., 2017, Schrock et al., 2009, Silberstein et al., 2003, Semenova et al., 2021). Moreover, previous evidence in CD patients suggests that propagation of theta-mu oscillatory activity from the GPi to the cortex contributes to CD pathophysiology (Chen et al., 2006, Neumann et al., 2012, Neumann et al., 2015). Given all the above considerations, ST may disengage the parietal-sensorimotor network from pathological oscillations propagating from the GPi (Tang et al., 2007, Chen et al., 2006, Silberstein et al., 2003). An alternative hypothesis is that theta desynchronization in the parietal-sensorimotor network mainly reflects cortical changes unrelated to subcortical abnormalities. Changes in parietal and sensorimotor cortex activation as measured by functional neuroimaging have been associated with ST execution (Naumann et al., 2000, Sarasso et al., 2020). Studies in CD patients suggested intrinsic neural circuit abnormalities in the parietal cortex and/or abnormalities in the connections between the parietal cortex and ipsilateral M1 circuits as compared to HCs (Porcacchia et al., 2014). Neuroimaging studies in patients with focal dystonia further demonstrated structural changes in the parietal cortex, specifically in regions functionally linked to the integration of sensory information (Zoons et al., 2011). The abnormal interaction between the PPC and motor and premotor areas suggests that the PPC plays a role in the pathophysiology of different forms of dystonia (Merchant et al., 2020, Thirugnanasambandam et al., 2020).

Abnormal sensorimotor integration has been hypothesized to play a major role in the pathophysiology of dystonia (Abbruzzese and Berardelli, 2003, Conte et al., 2019a). Several studies have shown that defective central processing of sensory inputs (Conte et al., 2014, Conte et al., 2019b, Avanzino et al., 2015) may lead to a mismatch between sensory input and motor output in dystonia (Desrochers et al., 2019, Manzo et al., 2022). The three experimental conditions (no touch, passive and examiner touch) simulating ST did not show any differences in post-touch EEG or EMG suggesting that tactile inputs, movement encoding, and proprioceptive afferents are all necessary components of ST. ST may therefore rely on the integration of sensory and motor neural processes (Lee et al., 2021). The observation that ST occurs only with a voluntary self-touch suggests that mechanisms of predicted sensory feedback (e.g., the output of a forward dynamics model) may also contribute to ST (Konczak and Abbruzzese 2013). We propose that encoding multiple sensory and motor neural processes associated with ST execution determines changes in parietal cortex activity, modulating sensorimotor integration encoding. In conclusion, our study suggests that ST-related “tactile stimulation” may reduce dystonic activity by modulating somatosensory and motor cortical processing via mechanisms of sensorimotor integration.

We acknowledge some limitations. For methodological reasons (i.e., the presence of the EEG cap and the intrinsic limitation of surface EMG in recording deep muscles, such as the splenius capitis), EMG activity was recorded only from bilateral SCM muscles. We enrolled only patients with clear laterocollis to limit possible confounding due to the heterogenic population. Future studies are needed to investigate how dystonic patterns other than laterocollis (i.e., torticollis, retrocollis, etc.) may influence our results. Finally, future studies integrating different techniques are needed to identify differences between CD patients with and without ST regardless of the execution of the maneuver, which may identify different disease subtypes.

## 5 Conclusions

In conclusion, our findings suggest that ST-induced effects on CD mainly depend on changes in the parietal-sensorimotor theta activity and sensorimotor alpha activity. ST may temporarily rebalance the excessive connectivity between parietal and sensorimotor cortices, restoring dysfunctional sensorimotor integration mechanisms in CD patients. The association between ST and desynchronization of parietal-sensorimotor theta activity and sensorimotor alpha activity may have translational therapeutic implications. Further studies using non-invasive brain stimulation are needed to ascertain whether the modulation of this oscillatory activity can induce behavioral improvements analogous to clinically effective ST.

## Funding

This research did not receive any specific grant from funding agencies in the public, commercial, or not-for-profit sectors.

## CRediT authorship contribution statement

**Nicoletta Manzo:** Conceptualization, Data curation, Investigation, Methodology, Project administration, Writing – original draft. **Giorgio Leodori:** Conceptualization, Formal analysis, Methodology, Project administration, Writing – original draft. **Giulia Ruocco:** Data curation, Investigation. **Daniele Belvisi:** Conceptualization, Supervision, Validation. **Shabbir Hussain I. Merchant:** Validation, Writing – review & editing. **Giovanni Fabbrini:** Validation, Writing – review & editing. **Alfredo Berardelli:** Conceptualization, Supervision, Validation, Project administration, Writing – review & editing. **Antonella Conte:** Conceptualization, Supervision, Validation, Project administration, Writing – review & editing.

## Declaration of Competing Interest

The authors declare that they have no known competing financial interests or personal relationships that could have appeared to influence the work reported in this paper.

## Data Availability

Data will be made available on request.

## References

[b0005] Abbruzzese G., Berardelli A. (2003). Sensorimotor integration in movement disorders. Mov. Disord..

[b0010] Amadio S., Houdayer E., Bianchi F., Tesfaghebriel Tekle H., Urban I.P., Butera C., Guerriero R., Cursi M., Leocani L., Comi G., Del Carro U. (2014). Sensory tricks and brain excitability in cervical dystonia: a transcranial magnetic stimulation study. Mov. Disord..

[b0015] Avanzino L., Tinazzi M., Ionta S., Fiorio M. (2015). Sensory-motor integration in focal dystonia. Neuropsychologia.

[b0020] Chen C.C., Kühn A.A., Trottenberg T., Kupsch A., Schneider G.H., Brown P. (2006). Neuronal activity in globus pallidus interna can be synchronized to local field potential activity over 3–12 Hz in patients with dystonia. Exp. Neurol..

[b0025] Cohen M.X. (2019). A better way to define and describe Morlet wavelets for time-frequency analysis. Neuroimage.

[b0030] Consky E., Lang A.E., Jankovic J., Hallett M. (1994). Therapy With Botulinum Toxin.

[b0035] Conte A., Rocchi L., Ferrazzano G., Leodori G., Bologna M., Li Voti P., Nardella A., Berardelli A. (2014). Primary somatosensory cortical plasticity and tactile temporal discrimination in focal hand dystonia. Clin. Neurophysiol..

[b0040] Conte A., Rocchi L., Latorre A., Belvisi D., Rothwell J.C., Berardelli A. (2019). Ten-Year Reflections on the Neurophysiological Abnormalities of Focal Dystonias in Humans. Mov. Disord..

[b0045] Conte A., Defazio G., Hallett M., Fabbrini G., Berardelli A. (2019). The role of sensory information in the pathophysiology of focal dystonias. Nat. Rev. Neurol..

[b0050] Desrochers P., Brunfeldt A., Sidiropoulos C., Kagerer F. (2019). Sensorimotor Control in Dystonia. Brain Sci..

[b0055] Foncke E.M.J., Bour L.J., van der Meer J.N., Koelman J.H.T.M., Tijssen M.A.J. (2007). Abnormal low frequency drive in myoclonus-dystonia patients correlates with presence of dystonia. Mov. Disord..

[b0060] Geng X., Zhang J., Jiang Y., Ashkan K., Foltynie T., Limousin P., Zrinzo L., Green A., Aziz T., Brown P., Wang S. (2017). Comparison of oscillatory activity in subthalamic nucleus in Parkinson’s disease and dystonia. Neurobiol. Dis..

[b0065] Grosse P., Edwards M., Tijssen M.A.J., Schrag A., Lees A.J., Bhatia K.P., Brown P. (2004). Patterns of EMG–EMG coherence in limb dystonia. Mov. Disord..

[b0070] Halje P., Brys I., Mariman J.J., da Cunha C., Fuentes R., Petersson P. (2019). Oscillations in cortico-basal ganglia circuits: implications for Parkinson’s disease and other neurologic and psychiatric conditions. J. Neurophysiol..

[b0075] Hutchison W.D., Dostrovsky J.O., Walters J.R., Courtemanche R., Boraud T., Goldberg J. (2004). Neuronal oscillations in the basal ganglia and movement disorders: evidence from whole animal and human recordings. J. Neurosci..

[b0080] Jinnah H.A., Hess E.J. (2018). Evolving Concepts in the Pathogenesis of Dystonia. Parkinsonism Relat. Disord..

[b0085] Kayser J., Tenke C.E. (2015). On the benefits of using surface Laplacian (current source density) methodology in electrophysiology. Int. J. Psychophysiol..

[b0090] Konczak J., Abbruzzese G. (2013). Focal dystonia in musicians: linking motor symptoms to somatosensory dysfunction. Front. Hum. Neurosci..

[b0095] Lee S.W., Cho H.J., Shin H.-W., Hallett M. (2021). Sensory tricks modulate corticocortical and corticomuscular connectivity in cervical dystonia. Clin. Neurophysiol..

[b0100] Leodori G., Fabbrini A., De Bartolo M.I., Costanzo M., Asci F., Palma V., Belvisi D., Conte A., Berardelli A. (2021). Cortical mechanisms underlying variability in intermittent theta-burst stimulation-induced plasticity: A TMS-EEG study. Clin. Neurophysiol..

[b0105] Manzo N., Ginatempo F., Belvisi D., Defazio G., Conte A., Deriu F., Berardelli A. (2022). Pathophysiological mechanisms of oromandibular dystonia. Clin. Neurophysiol..

[b0110] Merchant S.H.I., Frangos E., Parker J., Bradson M., Wu T., Vial-Undurraga F., Leodori G., Bushnell M.C., Horovitz S.G., Hallett M., Popa T. (2020). The role of the inferior parietal lobule in writer’s cramp. Brain.

[b0115] Naumann M., Magyar-Lehmann S., Reiners K., Erbguth F., Leenders K.L. (2000). Sensory tricks in cervical dystonia: perceptual dysbalance of parietal cortex modulates frontal motor programming. Ann. Neurol..

[b0120] Neumann W.-J., Huebl J., Brücke C., Ruiz M.H., Kupsch A., Schneider G.-H., Kühn A.A. (2012). Enhanced low-frequency oscillatory activity of the subthalamic nucleus in a patient with dystonia. Mov. Disord..

[b0125] Neumann W.-J., Jha A., Bock A., Huebl J., Horn A., Schneider G.-H., Sander T.H., Litvak V., Kühn A.A. (2015). Cortico-pallidal oscillatory connectivity in patients with dystonia. Brain.

[b0130] Neumann W.-J., Horn A., Ewert S., Huebl J., Brücke C., Slentz C., Schneider G.-H., Kühn A.A. (2017). A localized pallidal physiomarker in cervical dystonia. Ann. Neurol..

[b0135] Neuper C., Wörtz M., Pfurtscheller G. (2006). ERD/ERS patterns reflecting sensorimotor activation and deactivation. Prog. Brain Res..

[b0140] R. Oostenveld P. Fries E. Maris J.-M. Schoffelen FieldTrip: Open-source software for advanced analysis of MEG, EEG, and invasive electrophysiological data Comput Intell Neurosci. 2011 2011 1 9 156869. 10.1155/2011/156869 PMC3021840 21253357

[b0145] Patel N., Hanfelt J., Marsh L., Jankovic J. (2014). for the members of the Dystonia Coalition. Alleviating manoeuvres (sensory tricks) in cervical dystonia. J. Neurol. Neurosurg. Psychiatry.

[b0150] Pfurtscheller G., Klimesch W. (1991). Event-related desynchronization during motor behavior and visual information processing. Electroencephalogr. Clin. Neurophysiol. Suppl..

[b0155] Pfurtscheller G., Klimesch W. (1992). Functional topography during a visuoverbal judgment task studied with event-related desynchronization mapping. J. Clin. Neurophysiol..

[b0160] Pfurtscheller G., Lopes da Silva F.H. (1999). Event-related EEG/MEG synchronization and desynchronization: basic principles. Clin. Neurophysiol..

[b0165] Porcacchia P., Palomar F.J., Cáceres-Redondo M.T., Huertas-Fernández I., Martín-Rodríguez J.F., Carrillo F., Koch G., Mir P. (2014). Parieto-motor cortical dysfunction in primary cervical dystonia. Brain Stimul..

[b0170] Sarasso E., Agosta F., Piramide N., Bianchi F., Butera C., Gatti R., Amadio S., Del Carro U., Filippi M. (2020). Sensory trick phenomenon in cervical dystonia: a functional MRI study. J. Neurol..

[b0175] Schramm A., Reiners K., Naumann M. (2004). Complex mechanisms of sensory tricks in cervical dystonia. Mov. Disord..

[b0180] Schrock L.E., Ostrem J.L., Turner R.S., Shimamoto S.A., Starr P.A. (2009). The Subthalamic Nucleus in Primary Dystonia: Single-Unit Discharge Characteristics. J. Neurophysiol..

[b0185] Semenova U., Medvednik R., Popov V., Jinnah H.A., Shaikh A.G., Sedov A. (2021). Neuronal Activity of Pallidal Versus Cerebellar Receiving Thalamus in Patients with Cervical Dystonia. Cerebellum.

[b0190] Shakkottai V.G., Batla A., Bhatia K., Dauer W.T., Dresel C., Niethammer M., Eidelberg D., Raike R.S., Smith Y., Jinnah H.A., Hess E.J., Meunier S., Hallett M., Fremont R., Khodakhah K., LeDoux M.S., Popa T., Gallea C., Lehericy S., Bostan A.C., Strick P.L. (2017). Current Opinions and Areas of Consensus on the Role of the Cerebellum in Dystonia. Cerebellum.

[b0195] Silberstein P., Kühn A.A., Kupsch A. (2003). Patterning of globus pallidus local field potentials differs between Parkinson’s disease and dystonia. Brain.

[b0200] Tang J.K.H., Mahant N., Cunic D., Chen R., Moro E., Lang A.E., Lozano A.M., Hutchison W.D., Dostrovsky J.O. (2007). Changes in cortical and pallidal oscillatory activity during the execution of a sensory trick in patients with cervical dystonia. Exp. Neurol..

[b0205] Thirugnanasambandam N., Leodori G., Popa T., Kassavetis P., Mandel A., Shaft A., Kee J., Kashyap S., Khodorov G., Hallett M. (2020). Parietal conditioning enhances motor surround inhibition. Brain Stimul..

[b0210] Weinfurt KP, 1995. Multivariate analysis of variance, in: Grimm, L.G., Yarnold, P.R. (Eds.), Reading and understanding multivariate statistics. American Psychological Association, pp. 245–276.

[b0215] Wissel Jrg, Muller Jrg, Ebersbach G., Poewe W. (1999). Trick maneuvers in cervical dystonia: investigation of movement- and touch-related changes in polymyographic activity. Mov. Disord..

[b0220] Zoons E., Booij J., Nederveen A.J., Dijk J.M., Tijssen Ma.J. (2011). Structural, functional and molecular imaging of the brain in primary focal dystonia–a review. Neuroimage.

